# Simultaneous and extensive removal of the East Asian lithospheric root

**DOI:** 10.1038/s41598-020-60925-3

**Published:** 2020-03-05

**Authors:** Thomas C. Sheldrick, Tiffany L. Barry, Batulzii Dash, Chengshi Gan, Ian L. Millar, Dan N. Barfod, Alison M. Halton

**Affiliations:** 1 School of Geography, Geology and the Environment, University of Leicester, University Road, Leicester, LE1 7RH UK; 2 Department of Geology, Mongolian University of Science and Technology, Ulaan Baatar, 210646 Mongolia; 3 Guangdong Provincial Key Lab of Geodynamics and Geohazards, School of Earth Sciences and Engineering, Sun Yat-sen University, Guangzhou, 510275 China; 4 NERC Isotope Geosciences Laboratory, Keyworth, Nottingham, NG12 5GG UK; 5 NERC Argon Isotope Facility, Scottish Universities Environmental Research Centre, Scottish Enterprise Technology Park, East Kilbride, G75 0QF UK; 6 School of Physical Sciences, The Open University, Walton Hall, Milton Keynes, MK7 6AA UK

**Keywords:** Geochemistry, Geology, Petrology, Volcanology

## Abstract

Much evidence points to a dramatic thinning of East Asian lithosphere during the Mesozoic, but with little precision on when, or over what time scale. Using geochemical constraints, we examine an extensive compilation of dated volcanic samples from Russia, Mongolia and North China to determine when the lithosphere thinned and how long that process took. Geochemical results suggest that magmatism before 107 Ma derived from metasomatised subcontinental lithospheric mantle (SCLM), whereas after 107 Ma, melt predominantly derived from an asthenospheric source. The switch to an asthenospheric magma source at ~107 Ma occurred in both Mongolia and North China (>1600 km apart), whereas in eastern Russia the switch occurred a little later (~85 Ma). Such a dramatic change to an asthenospheric contribution appears to have taken, from beginning to end, just ~30 Myrs, suggesting this is the duration for lithospheric mantle weakening and removal. Subsequent volcanism, through the Cenozoic in Mongolia and North China does not appear to include any contribution from the removed SCLM, despite melts predominantly deriving from the asthenosphere.

## Introduction

**Figure 1:**
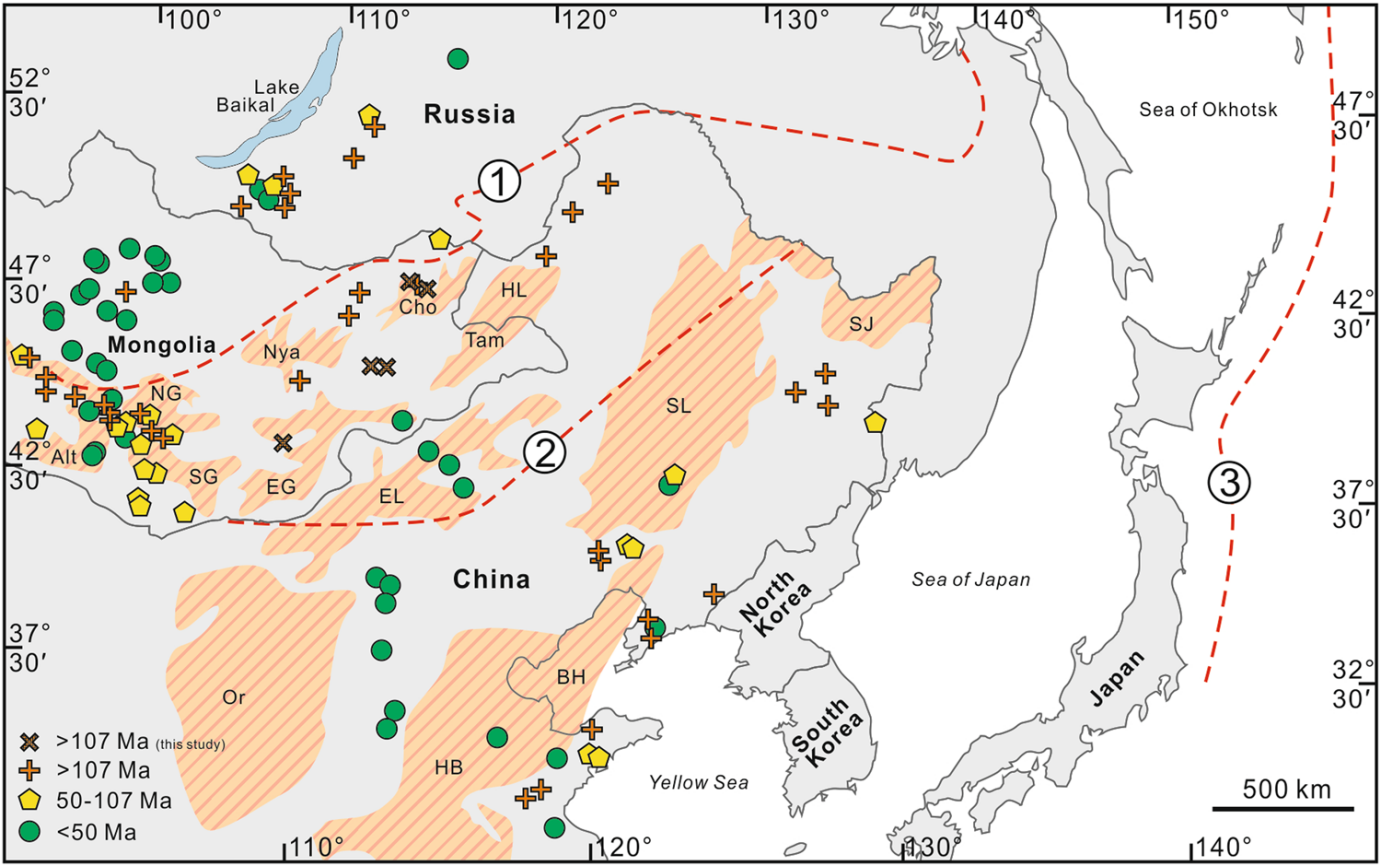
Map of Eastern Asia. Age distribution of Mesozoic and Cenozoic volcanic samples (Data sources available in Supplementary Material). Also shown is (1) the Mongol-Okhotsk Suture (remnant from the closure of the Mongol-Okhotsk Ocean), (2) Solonker Suture (remnant from the closure of the Paleo-Asian Ocean) and (3) Pacific plate active margin. Striped brown regions are sedimentary basins: NG, North Gobi; EG, East Gobi; Nya, Nyalga; Cho, Choibalsan. Tam, Tamtsag; HL, Hailar; Or, Ordos; HB, Huabei; BH, Bohai; EL, Erlian; SL, Songliao; SJ, Sanjiang. Crosses represent newly analysed samples for this study. The map was generated using CorelDRAW (www.coreldraw.com) and Google Earth (www.earth.google.com) software, with map data from: Google, Maxar Technologies.

Despite many studies on the widespread intraplate Mesozoic and Cenozoic magmatism across Eastern Asia^1–15^ (Fig. 1), little consensus has been reached about the cause of apparent lithospheric removal during the Mesozoic^6–11^, and intermittent follow-on magmatism^12–14^. Models for Mesozoic and Cenozoic volcanism in East Asia converge on themes of interactions between subducted slabs and upwelling mantle by either a cogenetic link with Paleo-Pacific slab roll-back^16–18^, convective erosion above either a subducting or stagnated slab e.g. associated with the Paleo-Asian^11^, Paleo-Pacific^19,20^ and/or Mongol-Okhotsk^15^^,^ oceans, or delamination^14–16^ due to thermo-chemical weakening of the lithospheric mantle. Studies advocating Mesozoic Paleo-Pacific slab roll-back have focused on magmatism in eastern Russia, eastern Mongolia^18^ and the North China Craton (NCC)^17^. However, assuming Mesozoic lithospheric removal in Eastern Asia was interrelated, such Paleo-Pacific slab roll-back models would struggle to explain such large-scale lithospheric removal in central Mongolia, >2000 km away from any active Pacific margin^16^. Furthermore, models advocating convective erosion above a Paleo-Pacific big mantle wedge^19,20^ would unlikely account for all the East Asian Mesozoic volcanism, because of the unrealistic extents required for flat-slab subduction^21^.

Despite the difficulties in discerning between any of the competing hypotheses for the cause of magmatism, instead, we look to place temporal constraints on the process, to improve our understanding of it. Here, we combine extensive existing data from Eastern Asia (see Supplementary Material), with new data from southern and eastern Mongolia, to constrain the timing of lithospheric removal (Fig. 1). We use melt compositions from dated samples only, from different volcanic fields across Eastern Asia, to evaluate source variation through time and space. Where geochemical data was not available for the dated sample, we have averaged data for the sample locality instead, with a data range given for the minimum and maximum values for each point/locality (see Supplementary Material; n = 459, where n =>370 have undergone radiometric dating). In addition to existing data, we present age constraints and geochemical data for 5 lavas from central and eastern Mongolia (Supplementary Fig. 1), that supplement this poorly constrained area (^40^Ar-^39^Ar ages between 171–132 Ma). We also provide new geochemical data for previously dated samples from Har Hotol, southern Mongolia^22^. Analytical procedures, including XRF, ICP-MS, Sr-Nd-Hf isotopes, and ^40^Ar-^39^Ar plateau diagrams, are included in the Supplementary Information (see Supplementary Material and Extended Data Table) along with a KMZ file which provides location information for all dated Mongolian samples utilised in this study.

### Far-reaching concurrent lithospheric removal

Melts derived from a metasomatised subcontinental lithospheric mantle (SCLM) are commonly characterised by depletion in some high-field strength elements (HFSE), such as Nb, Ta, Ti, and enriched isotopic signatures, compared to melts from asthenospheric mantle^23,24^. Therefore, such geochemical characteristics can be utilised to identify variations in the amount of metasomatised SCLM versus asthenospheric mantle^25^ input in volcanic samples from Eastern Asia.

**Figure 2:**
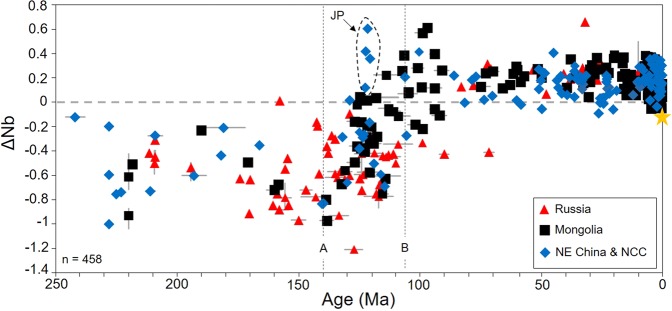
Time vs. ΔNb: geochemical variations through time and space for East Asian Mesozoic and Cenozoic volcanism. Where ΔNb = 1.74 + log (Nb/Y) −1.92 log (Zr/Y). Marker A = 140 Ma and Marker B = 107 Ma. JP = Jiaodong Peninsula; NCC = North China Caton; Yellow star = average MORB^25^. Data and sources reported in Supplementary Material. Where geochemical data was not available for the dated sample, we have averaged data for the sample locality instead, with a data range given for the minimum and maximum values for each point/locality (range bars).

A sensitive indicator of source characteristics is the expression ΔNb (where ΔNb = 1.74 + log (Nb/Y) −1.92 log (Zr/Y)); it is insensitive to the effects of mantle melting, source depletion by melt extraction, crustal assimilation or alteration processes^26^. Positive ΔNb values are consistent with a source from asthenospheric mantle or fertile lithospheric mantle that is not depleted in HFSEs; negative ΔNb values are consistent with a source depleted in Nb, such as a metasomatised SCLM. Assimilation-fractional crystallisation modelling^4,14^ and detailed petrological studies^2–4,8,12^ emphasise that crustal contamination was not a significant process in the genesis of the mafic volcanism included in this study. Most samples from Eastern Asia older than 107 Ma have negative ΔNb values (Fig. 2), signifying a dominantly metasomatised SCLM source. Melts younger than 107 Ma from Mongolia and the NCC have positive, or close to 0, ΔNb values, with Nb/La ratios >1 (Fig. 2; see Supplementary Material). Except for 122 My-old lamprophyres from Jiaodong Peninsula, which are associated with an area of localised rapid lithospheric removal (of the NCC)^17,27^, the general trend for samples between 140 Ma and 107 Ma is a gradual increase from negative to positive ΔNb values. This trend likely reflects a decrease in the involvement of the SCLM and crust, coupled with increasing asthenospheric input. We suggest this trend reflects a period of time (from 140 Ma to 107 Ma) when there was the greatest rate of metasomatised SCLM removal. Although all the data from Russia has negative ΔNb values for samples >107 Ma, two samples from the Khilok graben (Motninskoe), dated by K-Ar techniques^8,9^, have negative ΔNb values at 90 and 71.5 Ma. A lack of data (or magmatism) between 107–50 Ma makes it difficult to assess more generally, whether Russia underwent a change to positive ΔNb values around the same time as Mongolia, NE China and the NCC, or whether this actually occurred later. However, Russian Mesozoic samples from the Uda Sector (83–71 Ma), and Cenozoic volcanism has positive ΔNb values (Fig. 2) consistent with the data from elsewhere across the region.

**Figure 3:**
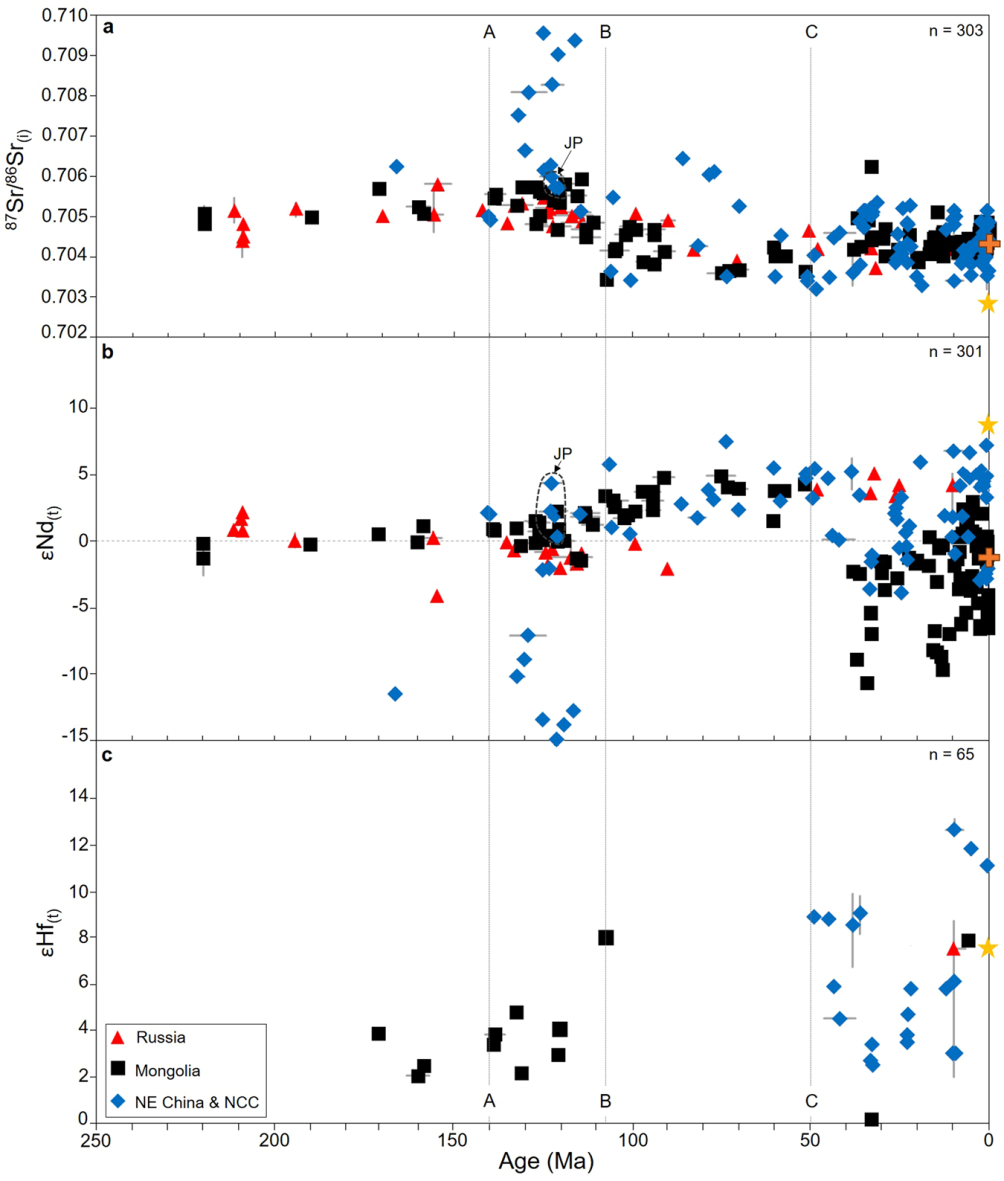
Time vs. Sr-Nd-Hf isotope plots for East Asian Mesozoic and Cenozoic volcanism. (**a)** Age vs. ^87^Sr/^86^Sr_(i)_. (**b)** Age vs. εNd_(t)._ (**c)** Age vs. εHf_(t)_. Marker A = 140 Ma, Marker B = 107 Ma and Marker C = 50 Ma. JP = Jiaodong Peninsula; NCC = North China Caton; Yellow star = average MORB^25^; Orange cross = representative average EM1 calculated from samples 47DS-8, 49DS-1, 51DS-1, 51DS-2 and PIT89-1^36,37^. (Value for εHf = −1.77. Not plotted, due to out of range of scale bar). Data and sources reported in Supplementary Material. Data uncertainty is within the size of the symbols, unless otherwise indicated. Where geochemical data was not available for dated samples, we have averaged data for the sample locality instead, with a data range given for the minimum and maximum values for each point/locality (range bars).

Evidence for increasing Mesozoic asthenospheric input between ~140 Ma and 107 Ma, across Eastern Asia, is supported by trends towards lower ^87^Sr/^86^Sr_(i)_, and increasing εNd_(t)_ and εHf_(t)_ (Fig. 3A; marker A-B). Detailed studies and numerical modelling, on sample specific locations, rule out extensive crustal assimilation processes prior to 107 Ma, with source composition being the dominant control on isotopic compositions^14,28,29^. We constrain a switch to dominantly asthenospheric magmatism at ~107 Ma (Fig. 3, marker B) in Mongolia and the NCC, by asthenospheric-like ^87^Sr/^86^Sr_(i)_ values of ~0.704, and εNd_(t)_ of ~5, likely signifying when the SCLM was finally removed. This asthenospheric geochemical signature persists until ~50 Ma (Figs. 2 and 3).

### Intermittent Cenozoic magmatism expose ‘ghost’ slabs in the upper mantle

From 50 Ma onwards (Fig. 3, marker C), Mongolian and NCC volcanic samples trend towards slightly higher ^87^Sr/^86^Sr_(i)_, highly variable εNd_(t)_ values between ~5 and −10, and variable εHf_(t)_ between 0 and 12 (though a paucity of data before 50 Ma make robust comparisons for εHf_(t)_ difficult). The trend to significantly lower εNd_(i)_, with only slightly lower εHf_(i)_ and slightly higher ^87^Sr/^86^Sr_(i)_ values, coupled with positive ΔNb values is best explained by incorporation of metasomatically-enriched material, either lithospheric mantle or ancient slab material, into an asthenospheric melt. The isotopic signature is very clear in Cenozoic samples from the Gobi Altai terrane (e.g., sample TB95-12.7.2 dated at 32.8 Ma, from the Sevrei Plateau, has εNd_(i)_ = −6.98 and εHf_(t)_ = 0.18), where there was also abundant magmatism from a SCLM source, at the end of the Mesozoic^4,13^. However, it is unlikely that the signature reflects crustal input, as demonstrated by assimilation-fractional crystallisation modelling^14^, and their positive ΔNb values (Fig. 2). It also does not appear to represent Mesozoic SCLM, as the isotopic signatures differ from those in the Mesozoic rocks (Figs. 2 and 3). Furthermore, it cannot represent post-Mesozoic, newly-replaced SCLM due to the wide-spread isotopic ratios that would require time to develop. Therefore, the geochemical signature points to an EM-1-like component, which, due to the positive ΔNb values and inferences from limited Mg isotope data^30^, suggest origins within the upper mantle. The most likely source for this EM-1-like component is therefore ancient slab material/fluids. Such material could be in the form of metasomatic fluids, but whether these fluids derive directly from a coherent slab, or are a component within otherwise upwelling mantle is unclear. It is likely that such fluids must originate from an ancient slab(s), rather than a more modern present day slab, due to the more extreme isotopic signatures.

### Towards a comprehensive model for East Asian magmatism

East Asia, as part of the Central Asian Orogenic Belt, underwent a complex Palaeozoic and Mesozoic tectonic history, with the involvement of multiple large-scale tectonic systems. These tectonic systems would have created a complex subsurface slab architecture which may have controlled upwelling mantle dispersal^31^ and assisted synchronous lithospheric removal. Whether a slab “graveyard” is driving intermittent Cenozoic magmatism needs to be considered further by utilising whole-mantle modelling^32^ with detailed geochemical studies to image and constrain slab dispersal. Such Mesozoic slabs have already been observed under Siberia^33^, and further south in East Asia^34^.

A recent review^35^ on the destruction of the NCC has attempted to place key time constraints on the possible relationship between the Paleo-Pacific plate, lithospheric thinning and magmatism. Four key time periods were identified: (1) an initial stage of low angle Paleo-Pacific flat subduction between ~170–145 Ma; (2) sinking/roll-back of the Paleo-Pacific slab and asthenospheric upwelling between 145–110 Ma; (3) the disappearance of the Paleo-Pacific slab into the lower mantle (110–55 Ma); and (4) the initiation of subduction of the present-day Pacific slab and associated formation of a big mantle wedge (<55 Ma). Interestingly, these timings correlate with changes in the geochemical signatures in Mongolia (Figs. 2 and 3), and possibly Russia, further supporting a shared geological link across the region. However, could the Paleo-Pacific tectonic system really be responsible for triggering Mesozoic and Cenozoic magmatism in Mongolia, so far away from the Pacific Plate active margin (Fig. 1)? Detailed numerical modelling is now needed to test how far magmatic upwellings can be triggered from the edge of the Paleo-Pacific flat-slab, during subduction and roll-back processes. Understanding what triggered the magmatism in Mongolia is likely to be a key to understanding magmatic processes across East Asia, and in fully understanding the destruction of the NCC.

Here, we show that the removal of SCLM and presence of asthenospheric volcanism at ~107 Ma under Mongolia, NE China and the NCC indicates a holistic process across the whole region. Whether such a dramatic switch to asthenospheric magmatism happened in Eastern Russia will require further data, especially good age constraints. Nevertheless, the removal of SCLM in Eastern Russia does appear to have occurred by at least 85 Ma. The cause of such wide-spread and synchronous removal of SCLM remains uncertain and vitally important to constrain for future understanding of the stability of continental lithosphere. Future studies should not be limited to localised conditions though and should consider the wider spatial constraints across the region.

## Supplementary information

Supplementary Material 1 - Analytical Methods.

Dataset 2 - Compiled Dataset.

Dataset 2 - Compiled Dataset.
