# Supplementary material for: Simultaneous and extensive removal of the East Asian lithospheric root
Source: Sci Rep. 2020 Mar 5;10:4128. doi: 10.1038/s41598-020-60925-3 (PMC7058094; doi:10.1038/s41598-020-60925-3)
Supplement: Supplementary file 1 — Supplementary Material 1 - Analytical Methods. [file 41598_2020_60925_MOESM1_ESM.pdf]

Supplementary Material

Simultaneous and extensive removal of the East Asian lithospheric root

Thomas C. Sheldrick[1*], Tiffany L. Barry[1], Batulzii Dash[2], Chengshi Gan[3], Ian L. Millar[4], Dan N. Barfod[5], Alison M. Halton[6]

[1] School of Geography, Geology and the Environment, University of Leicester, University Road, Leicester, LE1 7RH, UK

[2] Department of Geology, Mongolian University of Science and Technology, Ulaan Baatar 210646, Mongolia

[3] Guangdong Provincial Key Lab of Geodynamics and Geohazards, School of Earth Sciences and Engineering, Sun Yat-sen University, Guangzhou, 510275, China

[4] NERC Isotope Geosciences Laboratory, Keyworth, Nottingham, NG12 5GG, UK

[5] NERC Argon Isotope Facility, Scottish Universities Environmental Research Centre, Scottish Enterprise Technology Park, East Kilbride, G75 0QF, UK

[6] School of Physical Sciences, The Open University, Walton Hall, Milton Keynes, MK7 6AA, UK

Methodology

1.1 Major-element, trace-element and Sr-Nd-Hf analysis

Samples (TCS 18.1, 22.1, 25.2, 31.1, 33.1 and 56.1-56.5) were analysed for major- and trace-element abundance; and samples (TCS 18.1, 22.1, 25.2, 31.1, 33.1 and 56.5) for Sr-Nd-Hf isotope ratios (Extended Data Table 1). The whole-rock samples were crushed in a hardened steel fly press and then powdered in an agate mill; any amygdaloidal material was removed prior to crushing. Major-elements were determined on fusion beads made from pre-ignited powders which were fused with lithium metaborate flux (80% Li metaborate and 20% Li tetraborate) in a ratio of 1:5. Trace-elements were determined on 32 mm diameter pressed powder pellets (10g mixed with 7% PVA solution, as binding agent) to analyse for Rb, Ba, Co,*Th, *U, La, *Ce, *Nd *Sr, Ni, Cr, Pb, Cu, V, Zn, *Nb, La, *Y, and Zr. The analyses were conducted at the University of Leicester by X-ray fluorescence (XRF) spectrometry using a PANalytical ARL 8420 wavelength-dispersive system fitted with a Rh anode X-ray tube and a Philips PW1400 spectrometer with a W anode tube.

Trace, and rare-earth element concentrations (Th, U, Ce, Nd, Sm, Hf, Nb, Sr, Eu, Gd, Tb, Dy, Er, Y, Yb and Lu) were analysed by ICP-MS at the University of Leicester, on a Thermo iCAP-q quadrapole mass spectrometer using a standard HF-HNO_3 digestion method from powder. International standards BCR-1 (basalt) and JG-3 (granodiorite), as well as an in-house standard WS1-b (basalt) and blanks were run with the samples.

Whole-rock samples were analysed for Sr-Nd-Hf isotopes at NERC Isotope Geoscience Laboratory (NIGL), on 150-200 mg of sample. The powder was leached in 5 ml of 6M HCl at 60°C for 2 hours (to remove possible alteration phases) prior to addition of mixed ^{149}Sm-^{150}Nd and ^{176}Lu-^{177}Hf isotope tracers, and dissolution in HF-HNO_3. Primary columns containing Eichrom AG50x8 cation exchange resin were used to separate high-field-strength elements (HFSE), Sr and REE-rich fractions and Hf was separated from the HFSE fraction[1]. Neodymium was separated from the REE fraction using Eichrom LN-SPEC columns[2].

Strontium fractions were loaded onto outgassed single Re filaments using a TaO activator solution and analysed in a Thermo-Electron Triton mass spectrometer. Data are normalised to ^{86}Sr/^{88}Sr = 0.1194. NBS987 standard gave a value of 0.710260 ±0.000006 (9 ppm, 1-sigma, n=16). Sample data are normalised to a preferred standard value of 0.710250. Neodymium fractions were loaded onto one side of an outgassed double Re filament assembly using dilute HCl and analysed in a Thermo Scientific Triton mass spectrometer. Data are normalised to ^{146}Nd/^{144}Nd = 0.7219. JND-i standard gave a value of 0.512104 ±0.000004 (10.4 ppm, 1-sigma, n=17). BCR-2 rock standard gave ^{143}Nd/^{144}Nd = 0.512634 ±0.000004 (1-sigma, n=1).

Extended Data Table 1: Major and trace element geochemical data

Sample Number	TCS 18.1	TCS 22.1	TCS 25.2	TCS 31.1	TCS 33.1	TCS 56.1	TCS 56.2	TCS 56.3	TCS 56.4	TCS 56.5
SiO_2	65.10	55.93	56.17	56.20	61.87	52.75	51.16	52.42	52.13	52.68
TiO_2	0.80	1.52	1.77	2.04	1.34	2.91	2.97	2.92	2.86	2.86
Al_2O_3	15.25	16.85	16.33	16.20	15.13	14.49	14.83	14.60	14.49	14.54
$Fe_2O_{3(T)}$	4.84	7.80	8.71	8.42	6.13	10.42	8.79	9.86	10.57	10.13
MnO	0.075	0.215	0.078	0.078	0.067	0.125	0.128	0.162	0.164	0.144
MgO	0.75	2.26	2.17	1.72	1.93	3.53	2.45	3.44	3.68	3.56
K_2O	8.47	3.54	3.41	3.23	4.00	2.35	2.84	2.10	2.23	2.25
CaO	0.43	4.86	5.53	5.13	3.73	7.22	8.34	7.59	7.30	7.22
Na_2O_3	2.52	5.27	3.88	4.29	3.96	3.71	3.82	3.77	3.77	3.76
P_2O_5	0.130	0.812	0.980	1.079	0.689	1.641	1.820	1.641	1.707	1.625
LOI	1.56	1.15	1.00	1.46	1.17	0.80	2.34	1.34	1.03	0.94
Total	99.93	100.21	100.03	99.85	100.02	99.95	99.49	99.84	99.93	99.71
Co	BD	24.6	26.4	32.6	20.5	26.5	25.1	31.2	24.9	25.7
Ba	461.1	1618.8	1206.6	1108.5	1452.6	1359.7	1481.7	1353.9	1347.7	1380.7
Th	6.21	5.14	8.99	4.80	7.73	*5.72	*5.77	*5.0	5.85	5.71
U	1.43	1.22	1.49	1.32	1.87	*1.9	*2.15	*1.58	1.85	1.82
Ni	BD	17.3	18.6	31.3	22.0	19.4	21.8	27.1	20.3	20.5
Cr	-	34.7	70.9	78.2	34.7	28.5	26.1	33.3	27.2	34.8
Nb	13.53	18.60	20.37	16.65	28.54	*32.33	*38.67	*33.0	33.42	33.76
Cu	3.3	25	11.7	9.9	36.7	32.8	38.3	31.7	30.3	32.8
V	12.7	137.8	125.8	172.2	140.4	173.4	186.5	180.8	163.8	176.6
Zn	91.4	119.3	139.4	123.2	132.5	151.7	157.7	166.5	159.9	155.3
Rb	160.1	87.2	100.2	58.9	79.0	46.8	63.6	55.4	53.0	55.9
Sr	113.6	1320.2	903.4	1451.5	996.8	1211.3	1099.2	1222.1	1260.0	1249.4
Pb	27.2	17.7	21	18.4	20.5	20.2	21.2	18.8	19.7	20.1
Zr	871.1	362.8	475.7	340.4	513.8	546.8	646.5	546.7	545.7	558.7
Hf	4.64	7.69	10.13	6.13	10.08	-	-	-	-	12.11
Y	20.0	21.8	30.9	33.8	28.6	*38.0	*39.8	*38.3	40.8	39.7
La	54.9	66.3	76.6	55.2	82.0	88.3	105.1	83.4	83.6	88.1
Ce	59.8	124.1	151.1	118.5	161.1	*199.3	*241.6	*200.0	186.7	185.4
Nd	25.98	57.49	76.37	61.61	75.72	*101.89	*118.53	*101.44	103.36	103.11
Sm	4.75	9.32	12.43	10.76	12.53	-	-	-	17.60	17.12
Eu	1.02	2.46	2.73	2.58	3.10	-	-	-	4.29	4.25
Gd	3.66	6.60	8.79	7.93	8.97	-	-	-	12.50	12.52
Tb	0.58	0.86	1.15	1.13	1.16	-	-	-	1.67	1.62
Dy	3.15	3.98	5.42	5.65	5.34	-	-	-	7.16	7.18
Ho	0.62	0.67	0.94	1.04	0.85	-	-	-	1.19	1.19
Er	1.76	1.63	2.35	2.75	2.07	-	-	-	2.93	2.90
Yb	2.21	1.49	2.17	2.74	1.84	-	-	-	2.65	2.61
Lu	0.22	0.08	0.13	0.17	0.13	-	-	-	-	0.24

*Analysed by XRF; BD= Below Detection. Major elements as Wt.% and trace elements as PPM.

Isotope Data

Sample Number	TCS 18.1	TCS 22.1	TCS 25.2	TCS 31.1	TCS 33.1	TCS 56.5
$^{87}Sr/^{86}Sr$	0.713589	0.705501	0.705984	0.705978	0.705706	0.705976
$^{87}Sr/^{86}Sr_{(i)}$	0.708025	0.705071	0.705247	0.705692	0.705274	0.70573
$^{143}Nd/^{144}Nd$	0.512418	0.512595	0.512531	0.512563	0.512604	0.512536
$\varepsilon Nd_{(t)}$	-2.6	1.1	-0.1	0.5	1.0	-0.4
$^{176}Hf/^{177}Hf$	0.282793	0.282760	0.282748	0.282799	0.282842	0.282771
$\varepsilon Hf_{(i)}$	3.0	2.5	2.0	3.9	4.8	2.2

Hafnium fractions were dissolved in 1 ml of 2% HNO_3 + 0.1M HF, prior to analysis on a Thermo-Electron Neptune mass spectrometer, using a Cetac Aridus II desolvating nebuliser. Samples were introduced using 0.006 l/min nitrogen, in addition to Ar, to minimise oxide formation. Correction for ^{176}Yb was made using reverse-mass-bias correction of the $^{176}Yb/^{173}Yb$ ratio empirically derived using Hf mass-bias corrected Yb-doped JMC475

solutions[2]. ^{176}Lu interference was corrected using measured ^{175}Lu and assuming $^{176}Lu/^{175}Lu$ = 0.02653. Spike-stripping was carried out using an iterative algorithm. Data are reported relative to $^{179}Hf/^{177}Hf$ = 0.7325.

Hf standard JMC475 was analysed during and is reported relative to a value of 0.282160 for 176/177 standard[3], giving a mean $^{176}Hf/^{177}Hf$ value of 0.282146 ±0.000007 (1-sigma, n=11). Typical external precision was between 13 and 22 ppm.

Blanks for all elements were 50-80 pg with relative sample sizes of: Sr, 200 µg; Nd, 4 µg; and Hf, 2 µg.

1. 2 Argon dating

Five samples (TCS 18.1, 22.1, 25.2, 31.1 and 33.1) were selected for $^{40}Ar/^{39}Ar$ dating (Supplementary Figure 1). Samples were selected based on high total alkali (K_2O + Na_2O) content and the freshness of feldspar phenocrysts and groundmass material. Samples were crushed, washed in H_2O, but not acid leached, sieved to 300-600 µm and then carefully picked under a binocular microscope to sample the freshest groundmass separate.

Sample TCS 25.2 was analysed at The Open University (Milton Keynes, United Kingdom) and the other samples at NERC Argon Isotope Facility (AIF) at SUERC (Scottish Universities Environmental and Research Centre).

1.2.1 Sample analysis at The Open University

Prior to irradiation, at the McMaster Nuclear Reactor (McMaster University, Canada), sample TCS 25.2 was packaged in aluminium foil. Neutron flux was monitored using biotite mineral standard GA1550 (99.738 ±0.104, 1σ Ma)[4]. Standards were packed for irradiation, either side of the unknown sample and analysed using the single grain fusion method using a 1059 nm CSI fibre laser and a MAP215-50 mass spectrometer. The J values were then calculated by linear extrapolation between the 2 measured J values, and a 1% error on J is used.

The irradiated samples were loaded into an ultra-high vacuum system and a 1059 nm CSI fibre laser was focussed into the sample chamber and was used to step-heat the samples. After passing through a liquid nitrogen trap, extracted gases were cleaned for 5 minutes using 2 SAES AP-10 getters, one at 450°C and one at room temperature, following which the gases were let into a Nu Noblesse Instruments mass spectrometer for measurement. The mass discrimination value was measured at 295 for $^{40}Ar/^{36}Ar$ and system blanks were measured before and after every sample analysis. The system blanks measured before and after every one or two sample analysis were subtracted from the raw sample data. Results were corrected for ^{37}Ar and ^{39}Ar decay, and neutron-induced interference reactions. The following correction

factors were used: $(^{39}Ar/^{37}Ar)_{Ca}$ = 0.00065 ±0.00000325, $(^{36}Ar/^{37}Ar)_{Ca}$ = 0.000265 ±0.000001325, and $(^{40}Ar/^{39}Ar)_{K}$ = 0.0085 ±0.0000425; based on analyses of Ca and K salts. Ages were calculated using the atmospheric $^{40}Ar/^{36}Ar$ ratio of 298.56[5] and decay constants of Renne et al. (2011). Ages were calculated using Isoplot 3[6]. All ages are reported at the 2σ level and include a 1% error on the J value.

1.2.2 Sample analyses at the AIF

Prior to irradiation, the three samples (TCS 18.1, 22.1, 31.1 and 33.1) were packaged in copper foil and stacked in quartz tubes. The samples were irradiated at Oregon State University reactor in a cadmium-shielded facility (CLICIT). To monitor neutron flux and ^{39}Ar production, Fish Canyon sanidine (28.294 ±0.036 Ma, 1σ)[4] was used as the standard.

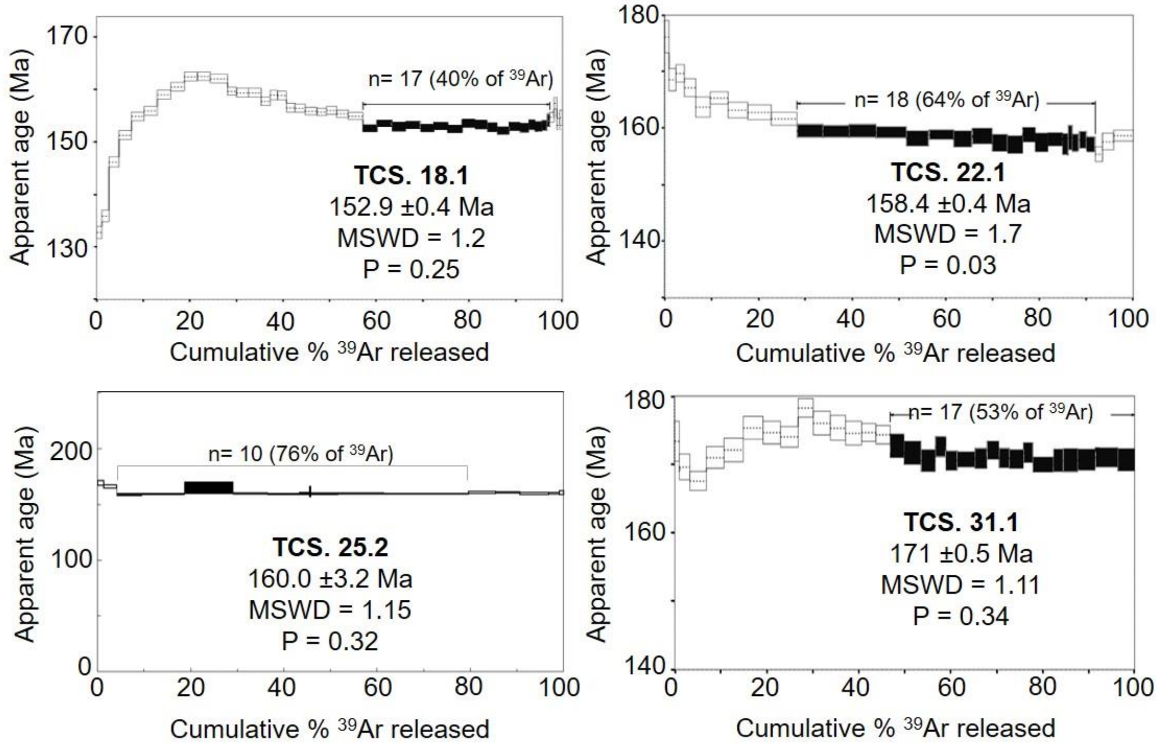

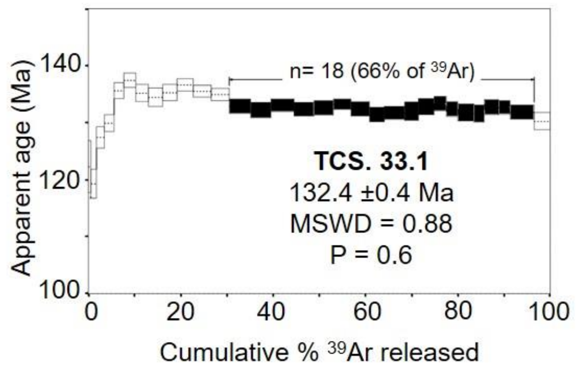

Sample number	Isochron age (Ma)
TCS. 18.1	154.2 ± 1.7
TCS. 22.1	158.2 ± 0.6
TCS. 25.2	161.7 ± 3.9
TCS. 31.1	171.4 ± 0.9
TCS. 33.1	132.5 ± 0.9

Supplementary Figure: ^{40}Ar-^{39}Ar age plateau diagrams. Also shown on each diagram is the MSWD (mean square weighted deviation) and P (Chi Squared value).

Gas was extracted from samples via step-heating using a mid-infrared (10.6 µm) CO_2 laser. The samples were housed in a doubly-pumped ZnS-window laser cell and loaded into a copper planchette containing nine 1 cm^2 square wells. Liberated argon was purified of active gases, e.g., CO_2, H_2O, H_2, N_2, CH_4, using three Zr-Al getters; one at 16°C and two at 400°C. Data were collected on a Mass Analyser Products MAP-215-50 single-collector mass spectrometer using an electron multiplier collector. The average total system blank for laser extractions, measured between each sample run, was $2.0\pm1.3\times10^{-15}$ mol ^{40}Ar, $5.7\pm4.6\times10^{-17}$ mol ^{39}Ar, $12.5\pm6.8\times10^{-18}$ mol ^{36}Ar. Mass discrimination was monitored daily, between and within sample runs by analysis of an air standard aliquot delivered by an automated pipette system. All blank, interference and mass discrimination calculations were performed with the MassSpec software package (MassSpec, version 8.058, authored by Al Deino, Berkeley Geochronology Center, Version 8.058).

References

(1) Münker, C., Weyer, S., Scherer, E. and Mezger, K., 2001. Separation of high field strength elements (Nb, Ta, Zr, Hf) and Lu from rock samples for MC-ICPMS measurements. Geochemistry, Geophysics, Geosystems, 2(12)

(2) Pin, C. and Zalduegui, J.S., 1997. Sequential separation of light rare-earth elements, thorium and uranium by miniaturized extraction chromatography: application to isotopic analyses of silicate rocks. *Analytica Chimica Acta*, *339*(1-2), pp.79-89.

(3) Nowell, G. and Parrish, R.R., 2001. Simultaneous acquisition of isotope compositions and parent/daughter ratios by non-isotope dilution-mode Plasma Ionisation Multi-collector Mass Spectrometry (PIMMS). Special Publication-Royal Society of Chemistry, 267(1), pp.298-310

(4) Renne, P.R., Balco, G., Ludwig, K.R., Mundil, R. and Min, K., 2011. Response to the comment by WH Schwarz et al. on “Joint determination of 40K decay constants and 40Ar∗/40K for the Fish Canyon sanidine standard, and improved accuracy for 40Ar/39Ar geochronology” by PR Renne et al.(2010). *Geochimica et Cosmochimica Acta*, *75*(17), pp.5097-5100.

(5) Lee, J.Y., Marti, K., Severinghaus, J.P., Kawamura, K., Yoo, H.S., Lee, J.B. and Kim, J.S., 2006. A redetermination of the isotopic abundances of atmospheric Ar. *Geochimica et Cosmochimica Acta*, *70*(17), pp.4507-4512.

(6) Ludwig, K.R., 2003. User's manual for IsoPlot 3.0. *A Geochronological Toolkit for Microsoft Excel*, *71*.
